# Predicting newborn birth outcomes with prenatal maternal health features and correlates in the United States: a machine learning approach using archival data

**DOI:** 10.1186/s12884-024-06812-5

**Published:** 2024-09-17

**Authors:** Robert D. Henry

**Affiliations:** Department of Psychology, Hope College, 35 E 12th St, Office 1159, PO Box 9000, Holland, 49422 MI USA

**Keywords:** Pregnancy, Birthweight, Head circumference, Emotion dysregulation, Machine learning

## Abstract

**Background:**

Newborns are shaped by prenatal maternal experiences. These include a pregnant person’s physical health, prior pregnancy experiences, emotion regulation, and socially determined health markers. We used a series of machine learning models to predict markers of fetal growth and development—specifically, newborn birthweight and head circumference (HC).

**Methods:**

We used a pre-registered archival data analytic approach. These data consisted of maternal and newborn characteristics of 594 maternal-infant dyads in the western U.S. Participants also completed a measure of emotion dysregulation. In total, there were 22 predictors of newborn HC and birthweight. We used regularized regression for predictor selection and linear prediction, followed by nonlinear models if linear models were overfit.

**Results:**

HC was predicted best with a linear model (ridge regression). Newborn sex (male), number of living children, and maternal BMI predicted a larger HC, whereas maternal preeclampsia, number of prior preterm births, and race/ethnicity (Latina) predicted a smaller HC. Birthweight was predicted best with a nonlinear model (support vector machine). Occupational prestige (a marker similar to socioeconomic status) predicted higher birthweight, maternal race/ethnicity (non-White and non-Latina) predicted lower birthweight, and the number of living children, prior preterm births, and difficulty with emotional clarity had nonlinear effects.

**Conclusions:**

HC and birthweight were predicted by a variety of variables associated with prenatal stressful experiences, spanning medical, psychological, and social markers of health and stress. These findings may highlight the importance of viewing prenatal maternal health across multiple dimensions. Findings also suggest that assessing difficulties with emotional clarity during standard obstetric care (in the U.S.) may help identify risk for adverse newborn outcomes.

**Supplementary Information:**

The online version contains supplementary material available at 10.1186/s12884-024-06812-5.

## Background

Predicting newborn health remains a critical factor for preventing neonatal mortality [1]. Newborn birthweight and head circumference are two robust markers of current and future risk of neonatal mortality, and both are collected nearly universally as growth benchmarks in the U.S. [2, 3]. Infants with low birthweight are at risk for neonatal death, infant health complications, and are even at higher risk for disease in adulthood (e.g., poor cardiovascular health) [4–8]. Additionally, newborn head circumference (HC) is related to mortality risk, brain volume, and is thus a marker of neurological development [9–12]. Pregnant individuals’ health features are in turn related to newborn outcomes because the developmental origins of health and disease can often be traced to the prenatal period [13–15]. Thus, newborn birthweight and HC may be a function of prenatal maternal health features and correlates [16].

Information about a mother’s environment can be relayed from parent to the developing fetus through a pregnant mother’s physiological signals, often via neuroendocrine, vascular, and epigenetic pathways [17–21]. For example, elevated cortisol and sympathetic nervous system activity may communicate to an unborn child that their mother lives in a highly stressful environment. Consequently, these features of a pregnant individual’s biology may prompt fetal neurodevelopmental changes, such as lower birthweight, preterm delivery, and greater distractibility in infancy [22, 23]. According to evolutionary theory, if the postnatal environment were to be aligned with the experience of the fetus in utero, these adaptations could help a child navigate stressful early-life circumstances (e.g., distractibility could help one mitigate risk of multiple threats) [24]. However, these neurodevelopmental changes can be detrimental in the long term, especially when the postnatal environment is misaligned with the one for which the fetus was adapting. This can result in increased risk for psychopathology, developmental delays, and neurocognitive deficits across the lifespan [21, 22, 25]. One challenge that researchers have encountered is that prenatal maternal health features and correlates are highly interrelated, making it difficult to choose which to include as independent variables in a model. Traditional approaches to removing predictors, such as ordinary least squares stepwise regression, are unable to do so without significantly inflating risk for Type I error and biased inferences [26].

### Machine learning models

As a first step toward eventually intervening to prevent the intergenerational transmission of risk, health professionals need to be able to efficiently predict newborn health as a function of readily available prenatal maternal health markers. One underutilized approach for predicting newborn birthweight and HC may be machine learning. Machine learning models are well-suited for robust prediction because they were designed to minimize prediction error and bias, and they can also test for nonlinear associations with relative ease [27, 28]. One class of machine learning, known as regularization, provides a way to examine effects of many associated predictors simultaneously. Regularized models shrink small predictor estimates toward zero, meaning that the most useful predictors emerge with the largest coefficients. This approach reduces risk of overfitting, which means that the final model has a higher chance of being replicated in independent samples. By minimizing prediction error due to overfitting, these types of machine learning models could provide unique information about the prenatal maternal health markers that most powerfully predict newborn birthweight and HC [26].

### Predictors of newborn outcomes

Markers of physical health (e.g., age and BMI), substance use (e.g., smoking status, alcohol use, and medication usage), and pregnancy-specific health markers (e.g., prior number of preterm births, abortions, and living children) are associated with newborn birthweight and HC in prior research [29–33]. Prenatal maternal mental health may also affect fetal growth and development. Numerous studies have highlighted links between prenatal maternal depression, anxiety, and dysregulated mood with risk for infant health complications, such as dampened vagal tone in response to stress [34–37]. Prenatal maternal emotion dysregulation may serve as a particularly useful predictor of infant outcomes because it is a transdiagnostic marker of adult mental health risk [36, 38]. Emotion dysregulation is defined as the over- and/or under-expression of affect that can interfere with goal-directed behavior and is often marked by emotional lability, difficulty with emotional clarity, and difficulty managing distress [38, 39]. Indeed, emotion dysregulation is a shared feature across numerous mood, personality, and substance use disorders, and it has several subcomponents that can make life challenging for individuals [40]. For pregnant individuals, emotion dysregulation has been associated with prenatal maternal psychopathology, stress, BMI, and cortisol as well as newborn neurobehavioral arousal and attention [41–46]. Emotion dysregulation could thus provide a useful way to understand risk for poor infant outcomes by capturing maternal mental health concerns that cut across a range of diagnoses [47].

Pregnant people’s mental and physical health is also shaped by their social and cultural context. Stress experienced during the prenatal period can “get under the skin” and alter fetal neurodevelopment [22]. Social determinants of health, such as socioeconomic disadvantage, crime exposure, and poverty, are often related to infant health at birth through biological embedding of stress during pregnancy [48–50]. For instance, an individual’s occupational prestige, a marker of resources and status, is associated with health and well-being [51]. Individuals with higher status jobs tend to also have greater opportunities for wealth accumulation and healthcare and are likely to have lower work-related stress. Prenatal social determinants of health can also explain racial/ethnic discrepancies in neonatal and maternal birth outcomes [52]. For example, in the United States, Black women tend to be at heightened risk for both maternal and infant mortality due to experiences with racism [53–55]. It may thus be crucial to examine race/ethnicity along with socioeconomic status when considering the developmental origins of newborn health and disease.

### Current study

HC and birthweight are both highly predictive of neonatal mortality risk [8, 56], and insufficient fetal growth is in turn associated with a greater probability of disease in childhood and later in life, including diabetes, cardiovascular disease, and psychopathology [21, 22, 25]. Thus, if healthcare providers could find prenatal maternal health features that predict newborn HC and birthweight, infant risk could eventually be better identified and prevented. HC and birthweight are the culmination of numerous, interrelated markers of prenatal maternal stress. Any prenatal maternal health feature or correlate may in turn be associated with hampered fetal growth. To determine if a newborn could be at risk for low birthweight or small HC, a healthcare professional could consider their mother’s physical health, psychopathology, and potential mediating social/contextual factors. Disentangling a causal chain between any one isolated marker of maternal and newborn health is likely to be ineffective and unhelpful. As such, the goal of this study is to determine which prenatal maternal health markers associate with newborn birthweight and HC. No published research to date has predicted newborn outcomes with facets of emotion dysregulation, physical health, and social determinants of health in the same model. In this study, we sought to use machine learning models that could have application and utility in obstetric care, helping professionals determine an infant’s risk prior to birth. Our primary aim was to determine which features and correlates of prenatal maternal health best predicted newborn birthweight and HC in a sample of pregnant people living in the western United States. Some aspects of these results may generalize to other parts of the U.S. as well as non-U.S. samples and could spur new international research in this area.

## Methods

### Participants

Our hypotheses, variables of interest, and analytic approach were pre-registered on the Open Science Framework (https://www.osf.io/f36ae/?view_only=c4ea6d46fb5c41869748cc2d1fb5fc30). The Difficulties in Emotion Regulation Scale (DERS) was used to determine eligibility for a separate longitudinal study in Salt Lake City, Utah, U.S.A. [45]. In this prior study, which consisted of a subsample from the current study’s larger sample, pregnant people were recruited such that those with high and low DERS scores were intentionally overrepresented. However, the current sample consisted of participants who did *and* did not qualify for the prior study due to DERS score. Thus, the current study’s sample should more closely represent the general population of pregnant people living in Salt Lake City and elsewhere in the U.S.

The current study thus consisted of an archival analysis using data collected previously. English- and Spanish-speaking women with singleton pregnancies (*N* = 594) were recruited during their prenatal appointments at Obstetrics & Gynecology clinics in the Salt Lake City, Utah area between January 2016 and October 2018. Recruitment flyers were also posted throughout the local community. Participants were eligible if they were between 18 and 40 years of age and in their third trimester of pregnancy. All participants provided written informed consent to complete a self-report measure of emotion dysregulation and for researchers to access their own and their newborn’s medical charts after delivery. The University of Utah Institutional Review Board approved all study procedures.

### Study design

The data used for this study were gathered for a prior study but had not yet been examined [45]. The data used for this study included medical chart records, self-reported DERS scores, and other demographic measures reported in recruitment (e.g., occupation).

### Measures

Emotion dysregulation was assessed during the third trimester of pregnancy with the DERS [57]. The DERS is a 36-item self-report questionnaire assessing emotion regulation problems. All items are on a 5-point Likert scale where 1 indicates *almost never* and 5 is *almost always*. In addition to a total score, the DERS has six subscales: *nonacceptance* of emotional responses; difficulty engaging in *goal*-directed behavior; *impulse* control difficulties; lack of emotional *awareness*; limited access to emotion regulation *strategies*; and lack of emotional *clarity*. Higher scores for all scales represent greater emotion dysregulation. Cronbach’s αs ranged from 0.78 to 0.95 for all scales, indicating acceptable to excellent internal consistency. Though discrete measures of psychopathology were not available in all 594 pregnant people, prior work that used a subsample of this study’s full sample of pregnant women found significant associations between emotion dysregulation and measures such as depression, anxiety, and borderline symptoms [45]. This supports the construct validity of the DERS in the current study.

The other 16 variables were extracted from participants’ medical charts. Predictors included both continuous and binary variables (see Table 1 for all 22 predictors). Race/ethnicity was coded as three binary variables: White and Non-Latina, Latina, and Non-White and Non-Latina. We coded race/ethnicity this way due to how our sample was distributed demographically and to avoid creating a large number of binary codes that were highly zero-inflated. Note that all participants in this sample self-identified as women; as such, we will use the term “Latina” rather than Latino/x to refer to participants who identified in this manner. Data were also coded for occupational prestige using a codebook of scores determined by the 2010 Census. Occupational prestige is a metric that is defined by how people typically perceive an occupation’s social standing, and is thus a product of perceived income, education, and other factors. Indeed, occupational prestige is a robust marker of socioeconomic status [58]. Two coders individually assigned codes using the codebook, and any discrepancies were resolved by a third coder. Participants who reported being unemployed were assigned prestige scores that were not reflected in the Census codes (at the median) because the coding system does not directly account for unemployment as an occupation. The two birth outcomes were newborn HC (centimeters) and birthweight (grams). Birthweights were standardized into z-scores relative to gestational age at delivery using nationally referenced norms [59].

**Table 1:** Sample characteristics

Variable	*n*	%	Mean	*SD*	Range
*Predictors*					
Age (years)^b^	547		29.08	5.06	18–40
Third-trimester BMI^b^	524		32.14	6.65	21.02–62.60
Psychotropic prescription^ab^	533	28.9			
Smoking status^b^	528				
Never smoker		85.0			
Former smoker		12.9			
Current smoker		2.1			
Alcohol use during pregnancy^ab^	523	17.4			
Number of preterm births^b^	525		0.20	0.60	0–6
Infant sex^ab^	534				
Male		48.7			
Female		51.3			
Number of abortions (spontaneous and otherwise)^b^	525		0.55	0.99	0–8
Number of living children^b^	524		1.18	1.26	0–10
Gestational diabetes diagnosis^ab^	534	4.9			
Pre-eclampsia diagnosis^ab^	533	3.9			
DERS (Total)^b^	590		73.96	22.90	36–155
Nonacceptance^b^	591		12.62	5.59	6–30
Goals^b^	591		12.58	4.57	5–25
Impulse^b^	591		10.56	4.36	6–29
Awareness^b^	592		13.16	4.41	6–28
Strategies^b^	591		15.58	6.55	8–40
Clarity^b^	592		9.42	3.25	5–23
Race/Ethnicity	560				
White & Non-Latina^ab^		60.2			
Latina^ab^		26.6			
All other races/ethnicities^ab^		13.2			
Occupational prestige^b^	594		41.34	26.57	0.00–97.05
*Outcomes*					
Standardized birthweight (z-scores)	528		-0.21	0.91	-2.58–2.58
Birthweight (grams)	530		3,316.75	482.11	1,310–4,570
Gestational age at birth (days)	540		273.58	9.41	232–293
Head circumference (cm)	465		34.40	1.66	28.5–39.0

*DERS* Difficulties in Emotion Regulation Scale

^a^Indicates the variable is dichotomous

^b^Indicates that the variable was used as a predictor for each outcome, for a total of 22 original predictors

### Analyses

Using R and the *caret* package [60], we followed a systematic machine learning approach to avoid overfitting and employed robustness checks and sensitivity analyses ([61]; see Supplementary Materials). Data were first preprocessed, during which predictors were centered and standardized. Missing predictor data were imputed via a bagged tree approach, in which each missing data point was modeled as a function of all other available variables in a decision tree. Trees were aggregated to arrive at final imputed datasets [62]. Our analytic plan was designed to maximize parsimony and interpretability. We began by removing unnecessary predictors using a Least Absolute Shrinkage and Selection Operator (LASSO) model, a type of regularized regression [26]. As previously noted, regularized regression models shrink parameter estimates toward zero that are non-contributory to prediction, thus maximizing the coefficient estimates of only contributory predictors. LASSOs take this approach and shrink superfluous estimates exactly to zero. Upon examining LASSO results, variables with zero-level coefficients were excluded from future rounds of modeling.

After running LASSO models to maximize parsimony, we then trained and tested a linear regularization ensemble to maximize prediction. This linear regression ensemble examined a range of coefficient penalization parameters to determine the best fitting model. The strictest penalization was equivalent to a LASSO model, whereas the most relaxed is referred to as a ridge regression (see Supplementary Materials for details). The model with the lowest root mean square error (RMSE) was deemed to be the best fitting model. To examine if a model was overfit, we calculated correlations between model predicted outcome values and original data for both training and testing data (*r*_train_ and *r*_test_, respectively) using *k*-fold cross-validation (where *k* varied by model, ranging from 3–10). We defined an overfit model as | *r*_train_ – *r*_test_ | ≥ 0.10, which would imply that a model had relatively poor out-of-sample performance. If regularized (linear) models continued to be overfit after multiple tuning attempts, we used a nonlinear model, specifically a Support Vector Machine (SVM). All final model features effects were then depicted using Individual Conditional Expectancy (ICE) plots and the R *iml* package [63]. ICE plots have many advantages, as they show what they model predicts across a range of hypothetical values for each participant. However, it is worth mentioning that they should not be interpreted like a raw data plot, as they depict the model’s expectations. In the ICE plots reported below, the full range of each outcome is *not* depicted because each model made a more conservative prediction as to the expected range of each outcome across feature values. We believe that this type of conservative modeling is more appropriate than risking extrapolating model findings to the most extreme values in our data.

## Results

Descriptive statistics are presented in Table 1. Demographic and health characteristics aligned with expected local population norms and newborn birthweight and HC were close to national averages [8, 59]. Approximately 11% (*n* = 60) of newborns were below the 10th percentile in terms of weight relative to gestational age, and approximately 24% (*n* = 111) were below the 10th percentile with respect to head circumference [2]. On average, our sample of pregnant adults reported low to moderate levels of emotion dysregulation (*M* = 74.0, *SD* = 22.9).

### Head circumference

The LASSO results indicated that 15 variables were non-contributory for predicting head circumference (HC). The seven predictors retained, all from medical chart records, were newborn sex, maternal preeclampsia status, BMI, number of preterm births, number of children, Latina race/ethnicity status, and psychotropic medication status. Thus, our model initially considered these the most effective linear predictors of newborn HC. We then ran additional regularized regression models to trim more unnecessary predictors and tune the model appropriately. The best model is reported in Table 2. Indeed, we found that a regularized regression was able to model HC most effectively.

**Table 2:** Best models, predictors, and performance

	**Head Circumference**	**Birthweight**
**Direction of Effect for Predictors**	Preeclampsia Status, ↓ *B* = -0.30	Occupational Prestige, ↑
	Male Newborn, ↑ *B* = 0.26	Number of Living Children, ~
	Number of Living Children, ↑ *B* = 0.24	Ethnicity (Non-White & Non-Latina), ↓
	Number of Preterm Births, ↓ *B* = -0.20	DERS-Clarity, ↑
	Ethnicity-Latina, ↓ *B* = -0.17	Number of Preterm Births, ~
	BMI, ↑ *B* = 0.12	-
**Model Type**	Ridge Regression (Linear)	Support Vector Machine (Nonlinear)
**Model Parameters**	λ = 0.3; α = 0	σ = 0.05;$C$= 0.1
**Model Performance**	*R*^2^ = 0.14	*R*^2^ = 0.08
	*RMSE = 1.59*	*RMSE = 0.86*
	*r*_training data, predictions_ = 0.40	*r*_training data, predictions_ = 0.30
	*r*_testing data, predictions_ = 0.37	*r*_testing data, predictions_ = 0.21

*DERS* Difficulties in Emotion Regulation Scale. Model parameters are explained in the manuscript. Arrows denote directional effects. Estimates (Bs) are listed for linear models only, as they are not calculated for nonlinear models. A ~ symbol denotes directional effects that are not clearly positive or negative (see Fig. 2)

**Fig. 1:**
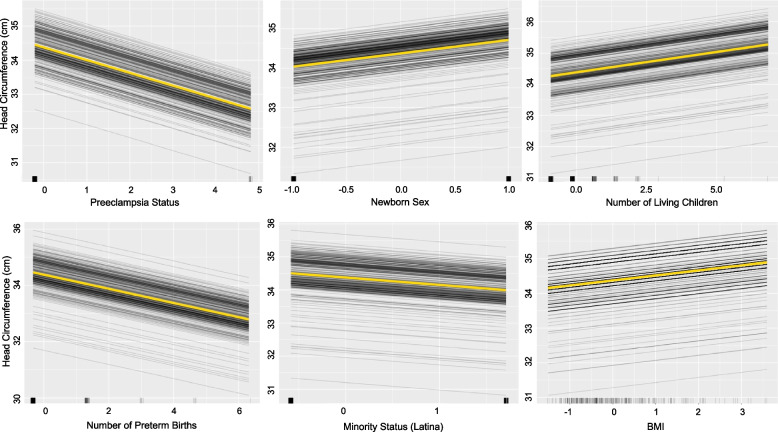
Plots Demonstrating Linear Effects in the Final Head Circumference Model. Each black line represents the predicted birthweight along potential values of a predictor for each participant*.* Yellow lines represent the average predicted head circumference. Tick marks along each x-axis indicate raw data. All predictors are centered and standardized. Because Preeclampsia Status, Newborn Sex (-1 = Female, + 1 = Male), and Minority Status (Latina) are dichotomous, y-values between the tick marks are not interpretable

Since this final model is linear, coefficient estimates are interpreted as they are in traditional regression modeling. However, because predictors were standardized, each coefficient represents the average increase in HC for every standardized unit increase in the predictor. This is an important step for regularized regression models, though it means that estimates for binary predictors are often uninterpretable. For binary predictors, we used ICE plots (Fig. 1) to approximate model-generated effects. The model predicted that a preeclampsia diagnosis would be associated with approximately a 2.5 cm decrease in newborn HC (compared to no diagnosis). Assigned male, compared to female, infants had approximately 0.6 cm larger HC. For every one standard deviation increase in the number of living children (*SD* = 1.26) and preterm births (*SD* = 0.60), the model predicted a 0.24 cm increase and 0.20 cm decrease in HC, respectively. Newborns of Latina participants tended to have 0.5 cm smaller heads than newborns of all other racial/ethnic groups. Finally, for a one standard deviation increase in a pregnant woman’s BMI (*SD* = 6.65), the model indicated a 0.12 cm increase in HC.

### Birthweight

**Fig. 2:**
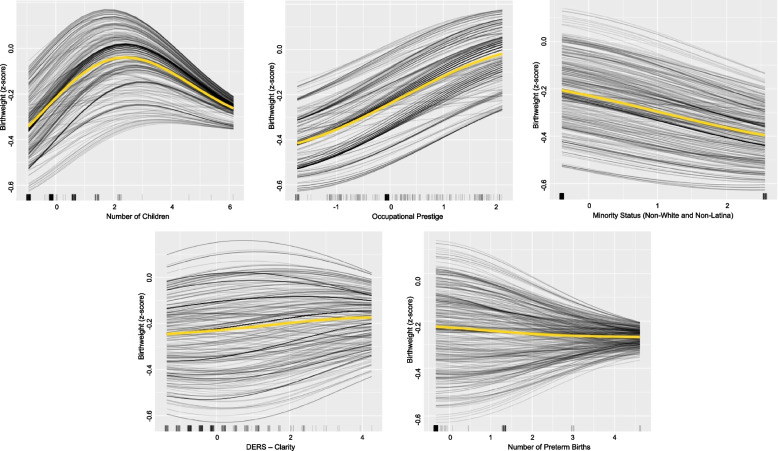
Individual Conditional Expectancy Plots for Predictors in the Final Birthweight Support Vector Machine Model. Top predictors of birthweight: occupational prestige, number of children. racial/ethnic minority status (Non-White and Non-Latina), DERS-Clarity, and number of preterm births. Each black line represents the predicted birthweight along potential values of a predictor for each participant*.* Yellow lines represent the average predicted birthweight (in z-score metric). Tick marks along each x-axis indicate raw data. All predictors are centered and standardized. Note that because Non-White and Non-Latina is dichotomous, birthweights between end values are not interpretable

Birthweight values are reported as z-scores (standardized by gestational age). For birthweight, the LASSO suggested removal of only three variables: maternal age, Latina racial/ethnic status, and DERS-Strategies. However, after several regularized ensembles, models were continuously overfit (i.e., *r*_train_ – *r*_test_ ≥ 0.10), suggesting that a nonlinear model could be warranted. Thus, we fit a radial kernel SVM to predict birthweight after LASSO feature selection. Several SVMs were run and tuned to arrive at top predictive performance (Table 2). The final SVM had five predictors: occupational prestige, number of living children, race/ethnicity (Non-White and Non-Latina), DERS-Clarity, and number of preterm births. Given that these SVMs are nonlinear, like above, we created individual conditional expectation (ICE) plots to approximate effects (Fig. 2), as regression coefficients do not exist for radial kernel SVMs.

The model predicted that as occupational prestige increased from our lowest to highest coded values, so did newborn birthweight by approximately 0.4 z-scores in a relatively linear fashion. The predicted association between number of children and birthweight was nonlinear. As number of prior children increased, expected birthweight increased initially. However, for participants with a relatively high number of children, birthweight was expected to decrease (Fig. 2). Next, we found a negative linear relation between birthweight and race/ethnicity (Non-White and Non-Latina). Birthweight was expected to drop by about 0.2 z-scores for Non-White and Non-Latina participants’ newborns, relative to all other participants (Fig. 2). Like number of children, DERS-Clarity and number of preterm births also had nonlinear associations with birthweight. Though the average expected relation between DERS-Clarity and birthweight was slightly positive, many participants were expected to have curvilinear relations. For some people, the model predicted that if they experienced more difficulties with emotional clarity, their newborn would have higher birthweights, though for many other people the opposite was expected (see Fig. 2). Thus, this relation is very complex and difficult to interpret meaningfully. On average, the effect of preterm births on expected birthweight was negligible (i.e., the average slope was nearly zero), though there were many individual differences. For several participants, as preterm births increased across the range of modeled values, the model expected birthweight to *decrease* by as much as 0.3 z-scores, and for many other participants, the model predicted an *increase* in birthweight beyond 0.3 z-scores.

## Discussion

The overarching goal of this study was to test robust, replicable, and interpretable machine learning models to predict newborn birth outcomes using information largely available in a typical maternal medical record. We modeled newborn HC and birthweight as functions of prenatal maternal health features and related correlates—including markers of physical health, pregnancy-specific health, and social determinants of health—that were gathered using an archival analysis of medical chart data as well as scores on a measure of emotion dysregulation. We achieved our study aims by first removing predictors that were non-contributory and then tuning model parameters to maximize predictive accuracy. Models were neither overfit nor underfit, indicating that they are relatively likely to replicate in out-of-sample testing [27].

First, with respect to head circumference (HC), a relatively simple linear model fit the data best (i.e., ridge regression). Moreover, the top predictors were infant sex and health markers specific to pregnancy (i.e., preeclampsia; preterm births; living children), replicating much of what has been documented in the literature [64–67]. It was somewhat surprising that preeclampsia status predicted HC, given how few of our participants were diagnosed with preeclampsia (*n* = 21). This may speak to the sheer strength of association in general between this medical condition and newborn HC. We also replicated prior findings in the literature with respect to BMI. Higher BMI was associated with larger HCs at birth, which may be due to larger placentas and greater fetal nutrition transfer [56].

Interestingly, race/ethnicity (Latina) was more contributory to HC prediction than many other health markers. For example, smoking, alcohol use, and gestational diabetes were excluded after the LASSO, meaning that the race/ethnicity (Latina) variable was more useful for prediction than these other potential predictors. Although many studies have documented obstetric and newborn health disparities among racial/ethnic minority individuals (compared to White individuals) [68–70], no published studies have linked self-identified Latina ethnicity with risk for relatively smaller neonatal head size. Infants born to Latina parents may be at greater risk for slower cranial growth due to unmeasured variables in our study, such as malnourishment and/or exposure to stress hormones, which may in turn be due to disparities in socioeconomic status, acculturation stress, and experiences with discrimination [71]. This finding could indicate that the race/ethnicity (Latina) variable functioned as a proxy for other aspects of risk, consistent with the social determinants of health hypothesis [48, 52]. Healthcare professionals can use this information to prioritize Latina individuals for intervention and prevention efforts, particularly if other potentially compounding risk factors are present (e.g., history of preterm births, preeclampsia). It is worth mentioning that Lorch and Enlow [50] note that racial/ethnic disparities in neonatal birth outcomes occur primarily on a systemic rather than an individual level. There is thus a critical need for public policy that addresses community-level health disparities in the U.S. This could be done by conducting large-scale interventions and obstetric risk assessments in neighborhoods with large Latina populations.

Interestingly, no facet of emotion dysregulation emerged as a predictor of newborn HC, nor did psychotropic medication use. This could suggest that either mental health is not related robustly to newborn head size, or that we simply did not collect data on the types of mental health markers that may predict fetal cranial growth [72]. Additionally, it is possible that psychotropic medication usage is related indirectly to newborn birth outcomes through mediating physiological mechanisms or via dosage-dependent effects that could not be examined in this cross-sectional archival study. One limitation of the current study is that we were not able to code specific psychotropic medication classes (e.g., antidepressants, mood stabilizers), nor did we have information on dosage and frequency of use, any of which could impact fetal growth.

A non-linear model was necessary to fit birthweight data. Each regularized model was overfit, indicating that linear models of birthweight are unlikely to generalize to other samples [27]. By employing a non-linear support vector machine (SVM), we bolstered the robustness and generalizability of our findings, though we did sacrifice a degree of interpretability due to the complexity of a radial kernel SVM. Nevertheless, our use of ICE plots made findings easier to understand. The strongest predictor of birthweight was occupational prestige, a coded variable associated with socioeconomic status and access to resources. The association between the two was positive, potentially because pregnant people in economically advantaged families have access to adequate nutrition and/or effective and regular healthcare. In contrast, less advantaged pregnant mothers likely have fewer health care and nutrition resources resulting in lower newborn birthweights [73]. Having a moderate number of living children was associated with higher birthweight compared to mothers with no or a small number of children, but having a high number of prior children was associated with decreasing birthweights. Nulliparous women are known to be at higher risk for low birthweight newborns, and in general, birthweight does increase with parity [29, 33]. However, individuals with higher parity (e.g., more than four births) can experience plateaus and even reductions in expected birthweight, relative to prior children, likely due to the fact that placental and intrauterine blood flow efficiency can only increase so much before eventually regressing to the mean [74–77].

Given that the prior two effects appear to replicate extant findings, there is reason to believe that the remaining effects may be similarly robust. The average relation between preterm births and birthweight was approximately zero, though individualized model projections suggest that some individuals may experience positive relations and others negative relations between these variables. This is surprising given that prior research has generally found that a history of preterm births increases risk for future preterm births—and generally lower birthweight [78]. We may have observed a different effect because we standardized birthweight by gestational age (as is considered best research practice) [79], indicating that although expected birthweight may decline with each prior preterm birth, subsequent infants may not necessarily be small relative to their gestational age. Additionally, the use of a nonlinear model (i.e., SVM) may also have allowed us to discover effects that have not yet been reported in the literature.

Women who were non-White and non-Latina also tended to have lower birthweight newborns. Though our coding approach to race/ethnicity was necessary for statistical purposes (given a small number of non-White and non-Latina participants), we recognize that, by doing so, this result becomes difficult to interpret as there is considerable heterogeneity among these participants. Nevertheless, this finding may also replicate prior findings indicating that African American and Asian American women may be at risk for low birthweight newborns [80, 81]. Indeed, racial/ethnic health disparities are well documented in the United States for people of color and other minoritized groups [6, 49, 50, 69]. Leading organizations such as the Society for Maternal–Fetal Medicine have articulated the importance of addressing systemic racism to combat these disparities in pregnant people and infants [82]. We are very cautious about generalizing these results to other cultures due to the relatively small sample size and homogeneity in participant geographic region. It is worth mentioning that there are decades of research demonstrating that there are health disparities in non-White pregnant people across the world [83–86]. Indeed, social determinants of health may have similar effects as documented in this study in other countries, e.g., China and Australia [87, 88]. A full discussion on the effects of prenatal maternal health on newborn outcomes by country of origin and race/ethnicity is beyond the scope of this paper. More research needs to be done to understand the complex and multifaceted associations between prenatal maternal race/ethnicity, social determinants of health, and newborn birth outcomes in the United States and other parts of the world.

Lastly, we found that difficulty with emotional clarity had nonlinear associations with birthweight (Fig. 2). Though the overall association between emotional clarity and birthweight was positive, for many the association was curvilinear (increasing then decreasing, or decreasing then increasing). The Clarity subscale of the DERS reflects the extent to which individuals understand their own emotions [57]. Extreme difficulty with emotional clarity, or alexithymia, is associated with psychopathology [89–91] and when experienced during pregnancy, alexithymia may predispose one to risk for future health problems [92, 93]. Kajanoja and colleagues [94] found that prenatal maternal alexithymia was linked to heightened risk for being overweight and having gestational diabetes, which could be explained by unhealthy diets, impulsive eating behavior (e.g., due to poor awareness that one is “full”), or HPA axis dysregulation. Relatedly, prenatal maternal obesity may link emotion dysregulation and cortisol levels [41]. This may in turn explain why greater difficulty with emotional clarity was, on average, predictive of greater birthweights. Thus, the inclusion of DERS-Clarity, a facet of overall emotion dysregulation, in our final model may indicate that this variable accounts for several underlying aspects of health and physiology (e.g., eating habits, exercise, cortisol). Alexithymia can be treated with mindfulness-based approaches, which help individuals attend to and define emotional states [95, 96]. This result emphasizes the importance of assessing a pregnant person’s emotional clarity. By doing so, clinicians may be able to use mindfulness-based interventions, perhaps by focusing on mindful eating and exercise behavior, to improve the health of pregnant individuals and their unborn children.

In sum, this study adds significantly to the maternal–fetal medicine literature. By using regularized regression models, it was possible to pit aspects of emotion dysregulation against established biomedical markers of fetal growth outcomes (e.g., prenatal maternal gestational diabetes, BMI) and social determinants of health. The fact that race/ethnicity emerged as a useful predictor of both newborn outcomes, and that difficulties with emotional clarity (a facet of emotion dysregulation) emerged as a predictor of birthweight suggests that these prenatal maternal characteristics need to be better understood in terms of the extent of their impact on fetal neurodevelopment and underlying mechanisms of action. Indeed, prior research has shown that prenatal maternal emotion dysregulation may predict newborn neurobehavior [46], and the current study indicates that emotion dysregulation may also predict newborn growth.

### Strengths and limitations

This study benefitted from several strengths. First, we relied on a structured and rigorous analytic plan. We pre-registered a series of machine learning models, beginning with feature selection, followed by regularized (linear) ensemble prediction, and, if need be, concluded with nonlinear prediction. This approach allowed us to detect nuances in the data and maximize interpretability of findings. For instance, using a linear model with fewer predictors for head circumference led to more interpretable results, which may make this model more useful or healthcare practitioners [97]. This also helped us account for potential collinearity issues that may have arisen. Second, all pregnant women completed the DERS and its subscales (a self-report measure of emotion dysregulation), a transdiagnostic index of mental health risk. This may be the first study to measure emotion dysregulation and compile prenatal-birth medical record data in a sample of over 500 pregnant people, and then use these features to predict fetal growth markers. Hopefully these findings spur additional research on these aspects of prenatal maternal wellbeing and their impact on the fetal environment.

However, this study also had limitations. First, we had a modest sample size for this type of analysis. Though we mitigated potential bias through several stringent model specifications (e.g., cross-validation, conservative over-fitting rules, sensitivity analyses), our sample size remains small for machine learning purposes and clinical utility. All participants were recruited from the same geographic region in the United States, and participants, on average, had relatively low overall emotion dysregulation. Thus, it would be unreasonable to assume that these results will generalize to pregnant people across the U.S. Though this sample was relatively diverse in terms of age, race/ethnicity, and socioeconomic status (i.e., occupational prestige), this sample is simply not heterogeneous enough to represent the entire country of pregnant people. We encourage caution when generalizing these results, as they represent a small but important step in establishing a link between prenatal maternal emotion dysregulation, social determinants of health, and newborn birth outcomes. It will be essential for larger, population-level studies to attempt to recreate these models and findings, in other parts of the country and especially in other parts of the world. Though these findings are replicable from a statistical perspective, this replicability is contingent on the next out-of-sample test being done on a group of people similar to the model’s training data. We want to stress that one should not assume these models will replicate in the exact same manner in different cultural groups or in other countries; they need to be validated in these contexts.

Second, we only examined main effects and did not consider moderation or meditation. Given the number of features and our structured, stepwise analytic approach, we chose to exclude potential interactions or mediation pathways in this project to simplify our models and maximize interpretability. Nevertheless, it is possible that meaningful effects could have been detected through moderation or mediation.

Third, we recognize significant limitations with our use of dummy coded variables, including alcohol use, psychotropic medication, and race/ethnicity. Given the data available in medical charts, we often did not have access to detailed information about prenatal substance use so we chose to simply code it in a dichotomous fashion. The lack of detailed information on pre-existing medical conditions and prenatal maternal health also limits our ability to fully describe and contextualize our sample. Our informed consent did not allow us to extract medical information beyond what was available at delivery, and in hindsight, we realize that detail on health status could have made the results even more impactful. We thus pre-registered use of dummy coding to maximize the amount of information available to us. For instance, we created three dummy codes for race/ethnicity to minimize the number of variables in our models and maximize the statistical ability of each race/ethnicity variable to predict the outcome of interest. Fourth, our health-related variables are cross-sectional and do not account for fluctuations over the course of pregnancy. For example, our alcohol use dummy code indicates whether a pregnant person drank *at all* over their pregnancy, meaning we do not know how often or how much they drank. This type of information would likely be more informative for understanding the developmental origins of health and disease. Lastly, we only modeled newborn HC and birthweight and chose not to use other outcomes. Our primary aim was to use variables that can be acquired from a single, standard medical chart and those that have regular use as markers of newborn development. It is possible that repeated measurements of fetal growth or neurobehavioral assessment scores at birth may be more robust indicators of neonatal well-being.

## Conclusions

We used a series of machine learning models to determine what aspects of prenatal maternal health predict newborn birthweight and HC, two markers of fetal growth and neurodevelopment. We found HC was predicted by markers associated with their mother’s prior pregnancy experiences (e.g., preeclampsia, aspects of parity/gravidity), as well as BMI. Latina women specifically also tended to have newborns with smaller heads, indicating a potentially socially-mediated risk factor. Newborn birthweight was also predicted by parity/gravidity, race/ethnicity (non-White and non-Latina women), and occupational prestige. Different aspects of racial/ethnic minority status could be associated uniquely with fetal growth and development. This finding replicates and extends upon prior literature, and should also be examined in larger samples. Yet, a highly novel finding of this paper was that birthweight was also predicted by a feature of emotion dysregulation. Indeed, greater difficulty with emotional clarity, a form of alexithymia, was associated with larger birthweights on average, though many person-specific effects were observed. Difficulty with emotional clarity may be correlated with health-related behaviors (e.g., impulsive eating, difficulty recognizing satiation). This finding suggests that mindfulness-based interventions could potentially be beneficial during the prenatal period, and that it may be important to assess aspects of emotion dysregulation, particularly difficulties with emotional clarity, to predict newborn birth outcomes. Clinicians may be able to use these findings to quickly and effectively identify women whose newborns may be at risk for restricted growth. By doing so, intervention and prevention efforts can begin prior to delivery, improving the lives of mothers and children.

## Supplementary Information

Supplementary Material 1

## Data Availability

Data for this project can be found at the following OSF Project Link: https://osf.io/we9a4/?view_only=b3f888f3da2e48068fa460bcebe13853.

## Abbreviations

HC: Head Circumference
BMI: Body Mass Index
DERS: Difficulties in Emotion Regulation Scale
LASSO: Least Absolute Shrinkage and Selection Operator
ICE: Individual Conditional Expectancy
SVM: Support Vector Machine
