# Supplementary material for: Predicting newborn birth outcomes with prenatal maternal health features and correlates in the United States: a machine learning approach using archival data
Source: BMC Pregnancy Childbirth. 2024 Sep 17;24:603. doi: 10.1186/s12884-024-06812-5 (PMC11409579; doi:10.1186/s12884-024-06812-5)
Supplement: Supplementary file 1 — Supplementary Material 1 [file 12884_2024_6812_MOESM1_ESM.docx]

**Supplementary Materials**

**Analytic Plan**

Outlier and exclusion criteria are detailed in the pre-registration. We anticipated outliers having minimal impact on regularization findings due to our high observation to parameter ratio, so we decided to employ a conservative approach to outlier removal (1). Raw data were inspected visually for outliers, and we also examined descriptive statistics and boxplots for each variable. Data points were only marked as outliers and removed if they were not feasible with respect to the rest of our sample. Unusual data points (e.g., BMI values over 50) were double-checked and confirmed.

See James and colleagues (2) for specifics on model fitting. Our analytic approach prioritized simplicity by first using regularization to remove unnecessary predictors and model each outcome with linear prediction (Supplementary Figure 1). We used nonlinear models only if linear models were ineffective. Preprocessing and imputation were completed with all 22 predictors included (and no outcomes). Missingness ranged from 0.2%–3.5% across all predictors. We used *k*-fold cross-validation and withheld test sets for LASSO models. Note that we do not report these model fit statistics or estimates because it is generally recommended that model performance not be interpreted when using a model primarily for feature selection (2). After running LASSOs, we then ran regularization ensembles, and we tuning penalty parameters throughout training (λ, range 0–2.5; α, range 0–1). When α equals 0, the model creates a ridge regression, 0.5 creates an elastic net, and 1 creates a LASSO; values between anchors denote a hybrid model. Given that there is no consensus definition of model overfitting, we used a relatively conservative overfitting rule to maximize out-of-sample performance and model generalization. We defined this rule in our pre-registered analytic plan.

If the best performing ensemble model (determined by the lowest Root Mean Square Error [RMSE]) was overfit, parameters were tuned, and the model was re-run. If ensemble models were overfit after several retuning attempts, we explored a nonlinear approach. We predicted birthweight with a Support Vector Machine (SVM). SVMs were trained and tested similar to regularized models. The SVM kernel function and corresponding boundary parameter (σ, range 0.001–0.05) and classification cost ($C$, range 0.1–1000). Best fitting SVM was chosen based on the lowest RMSE and if the overfit rule was violated. SVMs were tuned repeatedly to maximize performance.

**Sensitivity Analyses**

As described in the manuscript, we conducted several sensitivity analyses on our data as robustness checks. Note that because our training data samples were relatively small (i.e., around 300 observations), it was important to run these analyses, as well as account for random variation in analyses. With respect to head circumference data, we replicated the top performing model with listwise deletion, rather than imputation. Results were mostly unchanged. First, fit statistics (i.e., R^2^, RMSE) were virtually identical. Second, with the exception of BMI, all estimates were the same as the imputed model. Relative to results in Table 1, R^2^ dropped by 0.03, Δ*r* increased by 0.01, and RMSE increased by 0.04. The BMI estimate shifted from *B* = 0.10 in original analyses to *B* = 0.24 (a coefficient shift of 0.14), which may indicate that the BMI effect is less robust than the other effects. However, we also ran a second sensitivity analysis to further interrogate the BMI coefficient, whereby we used a series of different random start values throughout imputation and model fitting. This allowed us to determine the robustness of our findings and how much coefficients may change simply due to chance. The coefficient differences that emerged from random start values were all within 0.15, suggesting that the BMI coefficient change from the sensitivity value is within what can be expected from randomness.

With respect to the birthweight sensitivity analysis (i.e., listwise deletion on the top model), results were mostly unchanged as well. Fit statistics were virtually identical. Relative to results in manuscript Table 1, R^2^ dropped by 0.02, Δ*r* decreased by 0.03, and RMSE increased by 0.04. The only notable change that occurred was with respect to variable importance rankings—in the listed deleted model, number of living children was listed as the most important variable, and occupational prestige second most important (the reported model had these variables inverted). The second sensitivity analysis involving different random start values did not yield different results from the reported model. Overall, sensitivity analyses suggest that the findings are relatively robust (though the head circumference model may be less so).

A final sensitivity analyses was run to demonstrate how the machine learning model findings differed from a more traditional analytic approach: ordinary least squares (OLS) regression. As expected, the OLS model was highly collinear, and only three predictors emerged as statistically significant (Occupational Prestige [*b* = 0.004, *p* = .01], Smoking Status [*b* = -0.27, *p* = .008], and Number of Living Children [*b* = 0.16, *p* < .001]). A second OLS model was then run with only the top 5 predictors of birthweight retained (see Table 2). This model resulted in three significant predictors emerging (Occupational Prestige [*b* = 0.005, *p* = .002], Number of Preterm Births [*b* = -0.14, *p* = .04], and Number of Living Children [*b* = 0.12, *p* < .001]). This final OLS model had an adjusted R^2^ of 4.7%, almost half of the R^2^ from the final SVM model reported, likely due to the SVM’s ability to find nonlinear relations in the data. Thus, it is possible that a traditional OLS approach would increase risk for Type II error (false negative findings), and meaningful predictors of birthweight could be missed. Even for the variables which emerged in both the OLS and SVM, the OLS only depicts linear relations, whereas the SVM allows us to see nuance in these effects (see Figure 2). By harnessing machine learning rather than OLS regression, it was possible to first filter out the least robust predictors of newborn birthweight with regularization, and second demonstrate nonlinear effects of predictors of birthweight.

**Supplementary Figure 1**

*Complete Analytic Plan*

**
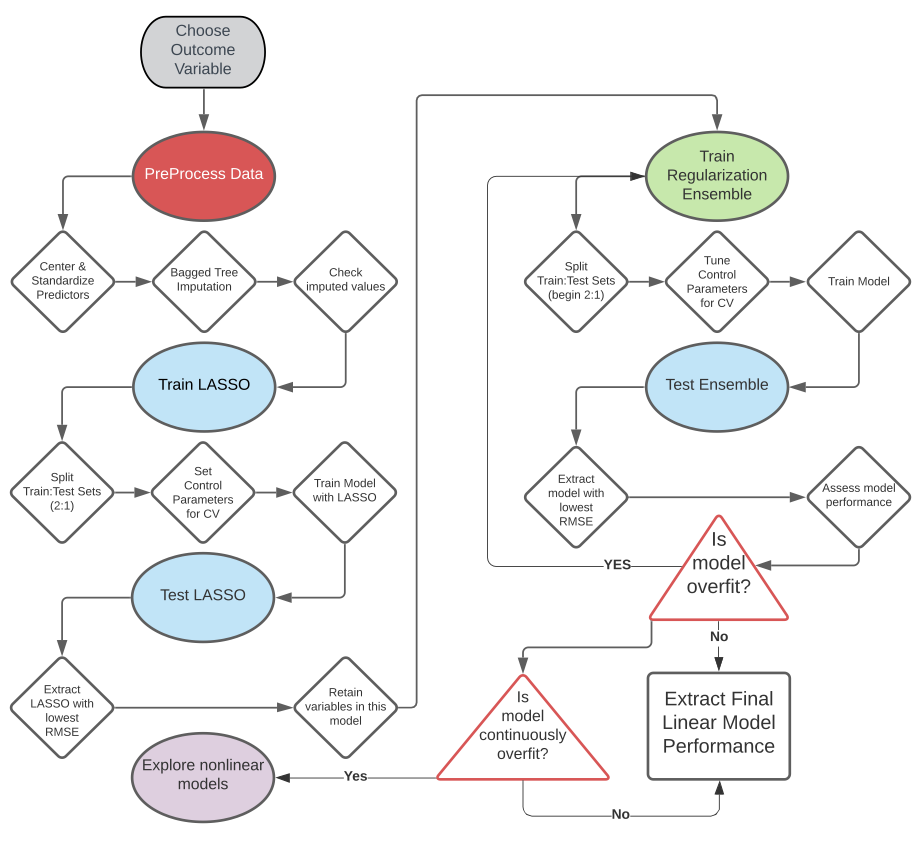
**

**Supplementary References**

1. Jeong J, Kim C. Effect of outliers on the variable selection by the regularized regression. Commun Stat Appl Methods. 2018;25(2):235–43.

2. James G, Witten D, Hastie T, Tibshirani R. An Introduction to Statistical Learning: with Applications in R [Internet]. 2nd ed. New York, NY: Springer; 2021 [cited 2024 Jun 13]. (Springer Texts in Statistics). Available from: https://link.springer.com/10.1007/978-1-0716-1418-1
